# Elevated Levels of Pro-Inflammatory Interleukin-6 in HIV Immunological Non-Responders Among the Indonesian Population

**DOI:** 10.3390/diagnostics15080959

**Published:** 2025-04-10

**Authors:** Agnes Rengga Indrati, Felicia Nathania Kosasih, Fitri Fadhilah, Amelia Pratiwi, Ummi Muthiah, Verina Logito, Anton Sumarpo, Jane Haryanto, Shofa Munaya, Ni Made Dwi Rosmiati, Dewi Kartika Turbawaty, Rudi Wisaksana

**Affiliations:** 1 Department of Clinical Pathology, Faculty of Medicine, Universitas Padjadjaran—Hasan Sadikin General Hospital, Bandung 40161, Indonesiaameliaphanailie@gmail.com (A.P.); ummi.muthiah@gmail.com (U.M.); verinalogito@gmail.com (V.L.); dwimadevip@gmail.com (N.M.D.R.); dewi.kartika@unpad.ac.id (D.K.T.); 2 Doctoral Program, Faculty of Medicine, Universitas Padjadjaran, Bandung 40135, Indonesia; fitrifadhilahssimkes@gmail.com; 3 Department of Clinical Pathology, Faculty of Medicine, Maranatha Christian University, Bandung 40164, Indonesia; anton.sumarpo@med.maranatha.edu; 4 Department of Internal Medicine, Faculty of Medicine, Universitas Padjadjaran—Hasan Sadikin General Hospital, Bandung 40161, Indonesia; rudi.wisaksana@unpad.ac.id

**Keywords:** cytokine, HIV, immunological non-responders, IL-6, IL-10

## Abstract

Approximately 10–35% of people living with HIV (PLHIV) on antiretroviral therapy (ART) fail to restore CD4^+^ T cell counts, a state known as immunological non-responder (INR) characterized by persistent immune activation and elevated cytokine levels. **Objective**: This study aimed to identify cytokines that can serve as biomarkers for immune activation and inflammation in INR patients. **Methods**: We conducted a cross-sectional study comparing two groups: INRs (PLHIV on ART with viral suppression) and immunological responders (IRs). We analyzed 40 samples of virologically suppressed PLHIV, measuring CD4^+^ T cell counts, viral load via RT-PCR, and cytokine levels through cytometric bead array (CBA). **Results**: The INR group exhibited significantly higher median serum levels of IL-6 (1.74 pg/mL vs. 0.94 pg/mL, *p* = 0.016) and IL-10 (1.65 pg/mL vs. 0.92 pg/mL, *p* = 0.03) compared to the IR group. **Conclusions**: Elevated IL-6 and IL-10 levels may serve as potential markers to distinguish INR from IR patients with areas under the curve (AUC) of 0.731 and 0.707, respectively.

## 1. Introduction

Antiretroviral therapy (ART) has significantly transformed the landscape of HIV management in which viral replication can be effectively suppressed. This remarkable advancement has not only facilitated the gradual restoration of CD4^+^ T cell counts, but has also contributed to a substantial decline in mortality rates among individuals living with HIV [1,2]. Despite these successes, a troubling subset of patients continues to grapple with immunological challenges, specifically the persistence of low CD4^+^ T cell counts, even in the presence of undetectable viral loads. Roughly 10–35% of individuals living with HIV fail to achieve normal CD4^+^ T cell levels, despite achieving viral suppression [3,4]. These individuals are referred to as immunological non-responders (INRs) or inadequate immunological responders [5,6]. INR patients are at greater risk of clinical deterioration, including opportunistic infections and non-AIDS events, such as nephropathy, metabolic syndrome, liver disease, cardiovascular disease, and cancer, and cognitive disorders related to HIV [7,8,9]. Clearly, the enduring immunological deficits experienced by INR patients underscore a critical aspect of HIV care that requires further exploration and understanding.

A key factor contributing to the ongoing health struggles of INR patients is persistent immune activation, which remains a major determinant of their clinical outcomes and a leading predictor of AIDS progression [5,10]. Factors contributing to this phenomenon include microbial translocation, residual viral replication, alterations in regulatory T cells (Tregs)/Th17 ratios, and co-infections [11,12,13]. Notably, while cytokine levels generally decline following the initiation of ART, certain pro-inflammatory cytokines have been observed to remain elevated in these individuals [5,12].

Excessive immune activation can precipitate a cascade of deleterious effects, including CD4^+^ T cell death, T cell exhaustion, and the development of lymphoid fibrosis. These processes collectively hinder the potential for CD4^+^ T cell recovery, exacerbating the immunological impairment seen in INR patients [6,11,12,13]. Given these complexities, this study aimed to elucidate the differences in cytokine profiles between HIV immunological non-responders and immunological responders (IRs). By identifying potential biomarkers of immune activation and chronic inflammation, this research sought to enhance our understanding of the underlying mechanisms that contribute to the unique challenges faced by INR individuals, ultimately informing more tailored and effective therapeutic strategies in HIV management and treatment.

## 2. Materials and Methods

### 2.1. Study Design and Participants

This comparative analytical observational study utilized a cross-sectional design and took place at Hasan Sadikin Hospital in Bandung, Indonesia, between September and December 2018. Sample collection utilized archived biological materials from the research center, with the sampling period spanning from May 2018 to May 2019. Eligible participants included adults living with HIV (PLHIV) aged 18 years or older who had been receiving antiretroviral therapy (ART) for a minimum of one year and maintained an undetectable viral load of less than 40 copies/mL. Participants were divided into two groups: the immunological non-response (INR) group, consisting of PLHIV on ART with viral suppression (viral load < 40 copies/mL) and CD4^+^ T cell counts of less than 350 cells/mm^3^; and the immunological response (IR) group, comprising PLHIV whose CD4^+^ T cell counts had risen to the normal range (≥350 cells/mm^3^). In addition, we decided to stratify based on the timing of ART initiation for PLHIV when their CD4^+^ T cell counts fell below 350 cells/mm^3^. Individuals with a history of autoimmune diseases or cancer were excluded from this study.

### 2.2. CD4^+^ Analysis

CD4^+^ cell counts were conducted utilizing the PIMA™ CD4^+^ assay (Abbott, Chicago, IL, USA). In summary, approximately 25 μL of venous blood was introduced into the PIMA™ CD4^+^ cartridge. For samples obtained via finger prick, the blood was applied directly to the cartridge. Following this, the PIMA™ CD4^+^ cartridge was promptly placed into the Alere PIMA CD4^+^ analyzer (Alere, Inc., Waltham, MA, USA), which automatically computed the CD4^+^ cell count.

### 2.3. Measurement of Viral Load

The viral load was evaluated using the Abbott m2000 real-time PCR system (Abbott, Chicago, IL, USA) on EDTA blood samples at the Research Unit of the Faculty of Medicine, Universitas Padjadjaran, Bandung.

### 2.4. Determination of Cytokine Levels

Serum cytokine levels were measured using the Cytometric Bead Array (CBA) method using the BD CBA Human Th1/Th2/Th17 Cytokine kit (catalog number 560484, BD Biosciences, Franklin Lakes, NJ, USA) based on the manufacturer’s protocols. In summary, 50 μL of a mixture containing captured beads, standard solutions, unknown samples, and detection reagents were added to each assay tube. These tubes then underwent a three-hour incubation at room temperature, shielded from light. Following this, samples were rinsed with 1 mL of wash buffer and centrifuged at 200× *g* for 5 min at room temperature. After removing the supernatant, the bead pellet in each tube was resuspended in 300 μL of wash buffer. The concentrations of cytokines in each sample were determined using the BD FACSLyric™ Flow Cytometry System (BD Biosciences, Franklin Lakes, NJ, USA) and analyzed with FCAP Array™ Software (version 3.0; BD Biosciences). The upper and lower detection limit was 5000 pg/mL and 0.01 pg/mL for all cytokines, respectively.

### 2.5. Statistical Analysis

The normality test was performed using SPSS statistical software (version 25, SPSS, Chicago, IL, USA). Data were analyzed using the non-parametric Mann–Whitney U test, with results presented as median values along with interquartile ranges (IQR). A *p*-value of less than 0.05 was considered as statistically significant. To determine a cut-off value for the cytokines, we generated a receiver operating characteristic (ROC) curve and area under the curve (AUC).

## 3. Results

### 3.1. Baseline Characteristics

A total of 70 datasets were collected and 40 subjects were included in this study: 15 in the HIV-INR group and 25 in the HIV-IR group. The median ages were 35 and 36 years for the INR and IR groups, respectively. Body mass index (BMI) was similar across groups (21.4 ± 2.4 vs. 21.4 ± 1.8). The median anti-retroviral therapy (ART) duration was 2 years for the INR group and 5 years for the IR group. All participants were treated with either zidovudine, lamivudine, lopinavir, ritonavir, nevirapine, efavirenz, emtricitabine, tenofovir, or a combination. Additionally, the duration of HIV diagnosis corresponded to the duration of antiretroviral therapy (ART), as ART initiation occurs shortly after a diagnosis is confirmed. The mean CD4^+^ T cell count in the INR group was 232.8 cells/µL, whereas the IR group exhibited a mean count of 570.2 cells/µL. Interestingly, the nadir CD4^+^ T cell count was significantly lower in the INR group (87.13 cells/µL) compared to the IR group (272.72 cells/µL). Moreover, co-infections were more prevalent in the INR group, particularly syphilis (36.4%). Notably, none of the coinfections identified in our study had been treated prior to data collection. Detailed baseline characteristics of study participants are presented in Table 1.

### 3.2. Cytokine Measurement

The comparison of IL-17A, IFN-γ, IL-4, IL-2, IL-6, and IL-10 levels between the HIV-INR and HIV-IR groups is summarized in Table 2. We observed a higher median level of cytokines in the HIV-INR group compared to the HIV-IR group, apart from IL-4.

**Figure 1:**
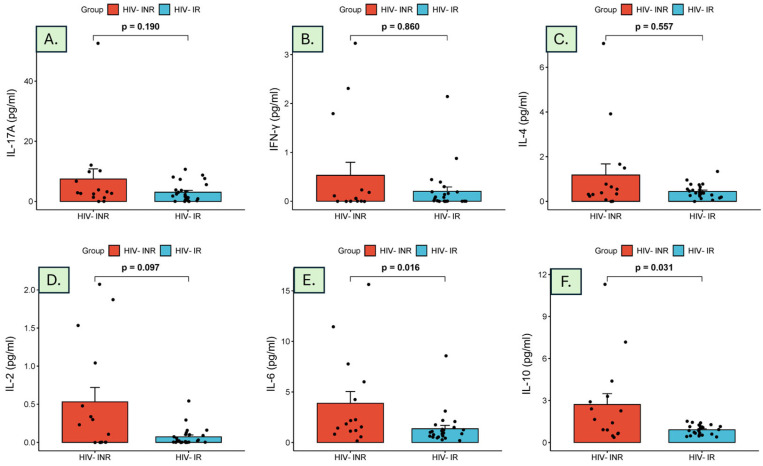
Distribution of cytokine levels among HIV-INR and HIV-IR groups: (**A**) IL-17A, (**B**) IFN-γ, (**C**) IL-4, (**D**) IL-2, (**E**) IL-6, and (**F**) IL-10 levels.

Moreover, the examination of cytokine levels revealed no significant differences in the levels of IL-17A, IFN-γ, IL-4, and IL-2 between the two groups. Interestingly, when the overall distribution pattern of all cytokines was visualized, the median serum levels of IL-6 and IL-10 were significantly elevated in subjects with HIV-INR compared to those with HIV-IR (Figure 1). Therefore, we evaluated the diagnostic effectiveness of IL-6 and IL-10.

### 3.3. Diagnostic Performance of IL-6 and IL-10

**Figure 2:**
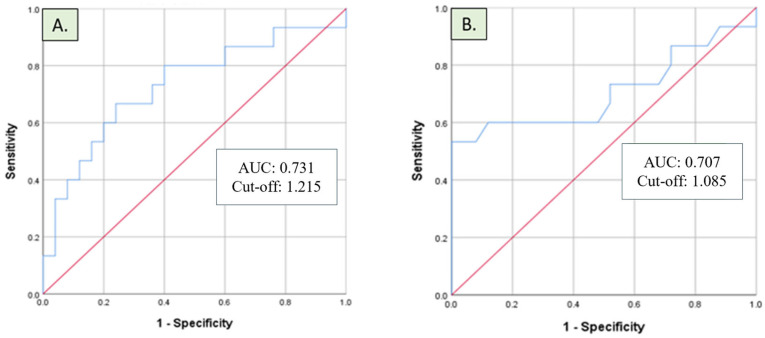
Receiver operating characteristic curves illustrating the diagnostic performance of (**A**) IL-6 and (**B**) IL-10 in distinguishing HIV immunological non-responders from immunological responders.

The diagnostic performance of IL-6 was assessed by the ROC curve, with an area under the curve (AUC) of 0.731 indicating moderate discrimination (Figure 2A). On the other hand, IL-10 levels were higher in INR patients (1.65 pg/mL vs. 0.92 pg/mL; *p* = 0.03), with an AUC of 0.707 (Figure 2B). The AUC of a ROC curve provides a measure of the discriminative ability of a diagnostic test. Therefore, an AUC of 0.731 for IL-6 and of 0.707 for IL-10 indicate fair discrimination, suggesting potential clinical utility for IL-6 and IL-10 as biomarkers for HIV-INR.

The optimal cut-off values and diagnostic performances of IL-6 and IL-10 are shown in Table 3. Our results indicated that with a cut-off value of 1.215 pg/mL, IL-6 achieved a sensitivity of 0.667 and a specificity of 0.360. On the other hand, IL-10 provided a sensitivity of 0.600 and a specificity of 0.360 with a cut-off value of 1.085 pg/mL to distinguish the HIV-INR group from the HIV-IR group.

## 4. Discussion

The demographic characteristics of our study indicated a predominance of males (80%) with a median age in the mid-thirties, which is consistent with data from previous reports [14,15]. Although we found no significant age difference between HIV immunological non-responders (INRs) and immunological responders (IRs) groups, other studies have reported that older age correlates with a higher incidence of INRs, potentially due to age-related changes in immunity and inflammation [16,17]. In our study, we defined the INR group as adults who had been on antiretroviral therapy (ART) for at least 12 months. A systematic review by Da Silva et al. indicated that the common cut-off points for ART duration in defining INR ranged from 12 to 24 months [18]. While an earlier cut-off can include individuals who may experience delayed immune responses, it also allows for earlier intervention, which could be more beneficial in managing these patients [1]. Significantly, the nadir CD4^+^ T cell count—the lowest recorded level since HIV diagnosis—was markedly lower in the INR group (87.13 ± 46.58 cells/µL) compared to the IR group (272.72 ± 160.52 cells/µL). This finding supports previous research, including studies by Lederman et al., Norris et al., and Noiman et al., all of which associated lower nadir CD4^+^ T cell counts with an increased incidence of INR [19,20,21]. Our data suggest that individuals with lower nadir counts may face ongoing immune activation and inflammation despite effective ART and viral suppression [5]. Furthermore, the higher prevalence of co-infections observed in the INR group—particularly syphilis, in 36.4% of cases—likely contributed to elevated immune activation, presumably by the interplay with regulatory T cells (Treg) and chronic inflammation [22,23,24].

This study illuminated the complex relationship between aberrant immune activation and the immune non-response (INR) in human immunodeficiency virus (HIV) patients undergoing antiretroviral therapy (ART). A key observation was that levels of soluble biomarkers, particularly pro-inflammatory cytokines, serve as crucial indicators of immune status in this population [25]. Notably, interleukin-6 (IL-6) emerged as a prominent biomarker due to its pivotal role in modulating immune responses and classification as a leading pro-inflammatory cytokine [26]. Our findings demonstrate that median IL-6 levels were significantly elevated in the INR group when compared to the immune response (IR) group. This observation is consistent with the work of Lederman et al., which established a similar association between elevated IL-6 levels and poor immunological outcomes in HIV-infected individuals [21]. Moreover, these results align with those of Hernández-Walias et al., who reported heightened IL-6 concentrations among patients exhibiting immune non-recovery compared to those with effective immunological responses [21]. The elevated levels of IL-6 in the INR group suggest an underlying chronic inflammatory state that may impair immune function. In addition, our results strongly support the hypothesis that persistent inflammation is detrimental to T cell functionality. This relationship is further corroborated by studies that illustrate how sustained pro-inflammatory signaling correlated with diminished T cell activity and responsiveness. Consequently, high IL-6 levels may not only serve as biomarkers for immune dysregulation, but also as potential therapeutic targets [27,28].

Moreover, our analysis revealed that interleukin-10 (IL-10) levels were significantly higher in the immune non-response (INR) group, averaging 1.65 pg/mL, compared to 0.92 pg/mL in the immune response (IR) group. This elevation in IL-10 is particularly concerning because it is associated with both immune activation and chronic inflammation, key factors that can undermine the immune system’s capacity to effectively combat HIV [29]. IL-10 is primarily recognized as an anti-inflammatory cytokine, and while it plays a role in modulating immune responses, its dysregulation can produce adverse effects, notably in the context of viral infections like HIV [30]. The role of IL-10 in HIV pathogenesis has been substantiated by previous studies, which highlighted its significant impact on immune function. Elevated levels of IL-10 can suppress the activation and proliferation of T cells, which are crucial for an effective immune response against HIV. This suppression can hinder the activity of cytotoxic T lymphocytes (CTLs) and the generation of memory T cells that are essential for long-term viral control [30,31]. Notably, higher levels of IL-10 have been linked with a larger HIV reservoir in both blood and lymph nodes among treated, aviremic individuals.

The diagnostic performance of IL-6 and IL-10 was rigorously assessed through our receiver operating characteristic (ROC) analysis, which yielded area under the curve (AUC) values of 0.731 for IL-6 and 0.707 for IL-10. The AUC serves as a critical metric in determining the accuracy of a biomarker. Hence, an AUC of 0.731 for IL-6 suggests a moderate ability to discriminate between immunological non-responders (INRs) and immunological responders (IRs). This finding is significant because it indicates that IL-6 levels can be utilized effectively in clinical practice to aid in the identification of INR patients, which is essential for improving treatment strategies and patient outcomes. The sensitivity and specificity values obtained for IL-6 suggest that nearly two-thirds of INR patients could be correctly identified based on their IL-6 levels, which speaks to its diagnostic relevance. Sensitivity is a crucial parameter in diagnostic tests, as it describes a test’s ability to correctly identify patients who have the condition—in this case, those who are INR. Given the importance of timely interventions for INR patients to prevent further complications, utilizing IL-6 as a biomarker could facilitate early identification and management, thereby improving health outcomes [31]. Conversely, the specificity associated with IL-6 was also noteworthy. In parallel, the analysis of IL-10 yielded sensitivity and specificity values of 60% and 36%, respectively. While IL-10 demonstrated potential as a complementary biomarker, these values indicate a more limited diagnostic utility compared to IL-6. The sensitivity of 60% suggests that although IL-10 can still aid in identifying a subset of INR patients effectively, it may not capture as high a proportion as IL-6. The relatively low specificity of 36% indicates that a significant number of patients may exhibit elevated IL-10 levels even in the absence of immunological non-response. This raises important considerations regarding the interpretation of IL-10 as a diagnostic marker [30,31,32]. Collectively, the diagnostic performance metrics for IL-6 and IL-10 highlight their distinct roles in the context of HIV-related immunological dysfunction. IL-6, with its combined moderate sensitivity and reasonable specificity, emerges as a more robust candidate for identifying INR patients. In contrast, while IL-10 supports the understanding of immune dysregulation in these individuals, its limited specificity necessitates caution in diagnosing and applying therapeutic interventions solely on the basis of elevated IL-10 levels [31,32].

However, there are several limitations to our study that should be addressed. First, the relatively small sample size may limit the generalizability of our findings to the broader population of PLHIV, particularly in diverse regions with differing epidemiological contexts. A larger sample size would strengthen the statistical power of our results and provide more reliable estimates of cytokine levels and their associations with immune response outcomes. In addition, larger sample sizes generally increase statistical power, making it easier to detect a true effect when it exists [33]. Second, the absence of detailed data on opportunistic infections and other co-infections, which could potentially influence IL-6 levels and contribute to the complexity of the immune response observed in INR patients, limits our ability to draw comprehensive conclusions. Future studies should incorporate a more extensive assessment of infectious co-morbidities in INR populations to elucidate their roles in immune activation. Additionally, our study design was cross-sectional, which restricted our capacity to establish causality. Longitudinal studies can help clarify the temporal relationship between cytokine levels, immune activation, and clinical outcomes in INR patients.

Finally, although we focused on IL-6 and IL-10 due to their established roles in immune activation, further research is warranted to explore the potential involvement of additional cytokines and biomarkers in the context of INR. Examination of their combined effects could yield valuable insights into the underlying mechanisms of immunological non-response. Nevertheless, our findings indicate that elevated levels of IL-6 and IL-10 serve as potential biomarkers for immune activation in HIV-INR patients. Monitoring CD4^+^ T cell counts in these individuals is essential even after achieving viral load suppression, as it can provide crucial insights into their immunological status and help guide therapeutic interventions.

This study highlighted that elevated median serum levels of IL-6 and IL-10 are significantly associated with HIV immunological non-responders compared to immunological responders. These findings suggest the potential use of IL-6 and IL-10 as biomarkers for immune activation in INR patients, emphasizing the necessity for ongoing immunological monitoring even after achieving viral suppression.

## 5. Conclusions

This study demonstrated that elevated levels of IL-6 and IL-10 are significantly linked to HIV immunological non-responders in comparison to those who respond immunologically. These findings highlight the potential utility of IL-6 and IL-10 as biomarkers for assessing immune activation in individuals who do not respond immunologically to therapy. This emphasizes the importance of continuous immunological monitoring, even after achieving viral suppression, to identify patients at risk of immunological non-response.

Furthermore, these biomarkers could be incorporated into clinical practice to guide treatment decisions and tailor therapeutic strategies for INR patients. Future research should focus on longitudinal studies to explore the relationship between IL-6 and IL-10 levels and clinical outcomes, as well as investigate potential interventions that could mitigate immune activation in this patient population.

## Figures and Tables

**Table 1:** Baseline characteristics of study participants.

Characteristic	HIV-INRs (*n =* 15)	HIV-IRs (*n =* 25)	*p*-Value
Age (years)			
Median (min–max)	35 (24–50)	36 (24–51)	0.695
Gender			
Male	15 (100.0%)	17 (68.0%)	0.014
Female	0 (0.0%)	8 (32.0%)	
Duration of ART (years)			
Median (min–max)	2 (1–10)	5 (1–16)	0.036
CD4^+^ T cells (cell/µL)			
Mean ± SD	232.8 ± 62.5	570.2 ± 191.9	<0.001
Nadir CD4^+^ T cells (cell/µL)			
Median (min–max)	85 (19–224)	232 (54–663)	<0.001
Body Mass Index (kg/m^2^)			
Mean ± SD	21.4 ± 2.4	21.4 ± 1.8	0.968
Coinfection	12 (80%)	13 (52%)	
Active Tuberculosis	2 (18.2%)	1 (5.9%)	0.087
Hepatitis B	2 (18.2%)	0 (0.0%)	
Hepatitis C	0 (0.0%)	4 (23.5%)	
Syphilis	4 (36.4%)	0 (0.0%)	
No coinfection	3 (27.2%)	12 (70.6%)	

HIV: human immunodeficiency virus; INRs: immunological non-responders; IRs: immunological responders.

**Table 2:** Concentrations of cytokine production.

Cytokine (pg/mL)	HIV-INRs (*n =* 15)	HIV-IRs (*n =* 25)	*p*-Value
IL-17A	2.87 (0.00–52.52)	1.74 (0.00–10.64)	0.190
IFN-γ	0.06 (0.00–3.23)	0.01 (0.00–2.14)	0.860
IL-4	0.39 (0.00–7.07)	0.39 (0.00–1.34)	0.557
IL-2	0.23 (0.00–2.07)	0.02 (0.00–0.54)	0.097
IL-6	1.84 (1.13–6.01)	0.94 (0.58–1.40)	0.016
IL-10	1.65 (0.36–11.30)	0.92 (0.40–1.51)	0.031

Data are presented as medians (IQRs); HIV: human immunodeficiency virus; INRs: immunological non-responders; IRs: immunological responders.

**Table 3:** Performance measurements and cut-off levels of IL-6 and IL-10.

Cytokine (pg/mL)	AUC	Cut-Off (pg/mL)	Sensitivity	Specificity
IL-6	0.731	1.215	0.667	0.360
IL-10	0.707	1.085	0.600	0.360

AUC: area under the curve.

## Data Availability

Data generated in this research are available upon request from the corresponding author.

## References

[1] Rb-Silva R., Goios A., Kelly C., Teixeira P., João C., Horta A., Correia-Neves M. (2019). Definition of immunological nonresponse to antiretroviral therapy: A systematic review. J. Acquir. Immune Defic. Syndr..

[2] Hernández-Walias F., Ruiz-de-León M.J., Rosado-Sánchez I., Vázquez E., Leal M., Moreno S., Vidal F., Blanco J., Pacheco Y.M., Vallejo A. (2020). New signatures of poor CD4 cell recovery after suppressive antiretroviral therapy in HIV-1-infected individuals: Involvement of miR-192, IL-6, sCD14 and miR-144. Sci. Rep..

[3] Gómez-Mora E., Massanella M., García E., Giles D., Bernadó M., Urrea V., Carrillo J., Ouchi D., Puig J., Negredo E. (2017). Elevated humoral response to cytomegalovirus in HIV-infected individuals with poor CD4+ T-cell immune recovery. PLoS ONE.

[4] Nakanjako D., Kiragga A.N., Musick B.S., Yiannoutsos C.T., Wools-Kaloustian K., Diero L., Oyaro P., Lugina E., Ssali J.C., Kambugu A. (2016). Frequency and impact of suboptimal immune recovery on first-line antiretroviral therapy within the International Epidemiologic Databases to Evaluate AIDS in East Africa. AIDS.

[5] Yang X., Su B., Zhang X., Liu Y., Wu H., Zhang T. (2020). Incomplete immune reconstitution in HIV/AIDS patients on antiretroviral therapy: Challenges of immunological non-responders. J. Leukoc. Biol..

[6] Taramasso L., Labate L., Briano F., Brucci G., Mora S., Blanchi S., Giacomini M., Bassetti M., Di Biagio A. (2023). CD4+ T lymphocyte recovery in the modern antiretroviral therapy era: Toward a new threshold for defining immunological non-responders. Front. Virol..

[7] Pacheco Y.M., Jarrin I., Rosado I., Campins A.A., Berenguer J., Iribarren J.A., Rivero M., Muñoz-Medina L., Bernal-Morell E., Gutiérrez F. (2015). Increased risk of non-AIDS-related events in HIV subjects with persistent low CD4 counts despite cART in the CoRIS cohort. Antivir. Res..

[8] Takuva S., Maskew M., Brennan A.T., Long L., Sanne I., Fox M.P. (2014). Poor CD4 recovery and risk of subsequent progression to AIDS or death despite viral suppression in a South African cohort. J. Int. AIDS Soc..

[9] Utay N.S., Hunt P.W. (2016). Role of immune activation in progression to AIDS. Curr. Opin. HIV AIDS.

[10] Appay V., Kelleher A.D. (2016). Immune activation and immune aging in HIV infection. Curr. Opin. HIV AIDS.

[11] Bruzzesi E., Sereti I. (2018). Residual immune activation and latency. Curr. Top. Microbiol. Immunol..

[12] Hileman C.O., Funderburg N.T. (2017). Inflammation, immune activation, and antiretroviral therapy in HIV. Curr. HIV/AIDS Rep..

[13] Burgos-Ramos E., Martos-Moreno G.Á., Argente J., Barrios V. (2012). Multiplexed Bead Immunoassays: Advantages and Limitations in Pediatrics.

[14] Thompson C.G., Gay C.L., Kashuba A.D.M. (2017). HIV persistence in gut-associated lymphoid tissues: Pharmacological Challenges and Opportunities. AIDS Res. Hum. Retroviruses.

[15] Maskew M., Brennan A.T., Westreich D., McNamara L., MacPhail A.P., Fox M.P. (2013). Gender differences in mortality and CD4 count response among virally suppressed HIV-positive patients. J. Womens Health.

[16] Li C.X., Li Y.Y., He L.P., Kou J., Bai J.S., Liu J., Tian B., Cao L.J., Wang K.H., Kuang Y.Q. (2019). The predictive role of CD4+ cell count and CD4/CD8 ratio in immune reconstitution outcome among HIV/AIDS patients receiving antiretroviral therapy: An eight-year observation in China. BMC Immunol..

[17] Celerino da Silva R., Alves N.M.P., Gondim Silva M.L., Agrelli A., Coelho A.V.C., Guimarães R.L., Arraes L.C., Crovella S., Brandão L.A.C. (2021). Brief report: Polymorphisms in TNF-α/TNFR1 pathway genes are associated with CD4+ T-cell recovery in HIV-1-infected individuals on antiretroviral therapy. J. Acquir. Immune Defic. Syndr..

[18] Noiman A., Esber A., Wang X., Bahemana E., Adamu Y., Iroezindu M., Kiweewa F., Maswai J., Owuoth J., Maganga L. (2022). Clinical factors and outcomes associated with immune non-response among virally suppressed adults with HIV from Africa and the United States. Sci. Rep..

[19] Keating S.M., Golub E.T., Nowicki M., Young M., Anastos K., Crystal H., Cohen M.H., Zhang J., Greenblatt R.M., Desai S. (2011). The effect of HIV infection and HAART on inflammatory biomarkers in a population-based cohort of women. AIDS.

[20] Lederman M.M., Calabrese L., Funderburg N.T., Clagett B., Medvik K., Bonilla H., Gripshover B., Salata R.A., Taege A., Lisgaris M. (2011). Immunologic failure despite suppressive antiretroviral therapy is related to activation and turnover of memory CD4 cells. J. Infect. Dis..

[21] Bono V., Augello M., Tincati C., Marchetti G. (2022). Failure of CD4+ T-cell recovery upon virally-effective cART: An enduring gap in the understanding of HIV^+^ immunological non-responders. N. Microbiol..

[22] Chen L., Bao D., Gu L., Gu Y., Zhou L., Gao Z., Huang Y. (2018). Co-infection with hepatitis B virus among tuberculosis patients is associated with poor outcomes during anti-tuberculosis treatment. BMC Infect. Dis..

[23] Rivera M.M., Soza A., Jazwinski A., Mi L., Kleiner D.E., Zhao X., Zuber C., Brust D., Hsu E., Simpson J. (2015). HIV through the looking glass: Insights derived from Hepatitis B. J. Acquir. Immune Defic. Syndr..

[24] Hattab S., Guiguet M., Carcelain G., Fourati S., Guihot A., Autran B., Caby F., Marcelin A.G., Costagliola D., Katlama C. (2015). Soluble biomarkers of immune activation and inflammation in HIV infection: Impact of 2 years of effective first-line combination antiretroviral therapy. HIV Med..

[25] Luo Y., Zheng S.G. (2016). Hall of fame among pro-inflammatory cytokines: Interleukin-6 gene and its transcriptional regulation mechanisms. Front. Immunol..

[26] Shive C.L., Freeman M.L., Younes S.A., Kowal C.M., Canaday D.H., Rodriguez B., Lederman M.M., Anthony D.D. (2021). Markers of T cell exhaustion and senescence and their relationship to plasma TGF-β levels in treated HIV+ immune non-responders. Front. Immunol..

[27] Zicari S., Sessa L., Cotugno N., Ruggiero A., Morrocchi E., Concato C., Rocca S., Zangari P., Manno E.C., Palma P. (2019). Immune activation, inflammation, and non-AIDS co-morbidities in HIV-infected patients under long-term ART. Viruses.

[28] Dos Santos Guedes M.C., Carvalho-Silva W.H.V., Andrade-Santos J.L., Brelaz-de-Castro M.C.A., Souto F.O., Guimarães R.L. (2023). Thymic exhaustion and increased immune activation are the main mechanisms involved in impaired immunological recovery of HIV-positive patients under ART. Viruses.

[29] Naidoo S.J., Naicker T. (2024). The enigmatic interplay of interleukin-10 in the synergy of HIV infection comorbid with preeclampsia. Int. J. Mol. Sci..

[30] Indrati A.R., Sumarpo A., Atmadja P., Wisesa R.R., Ghozali M., Judistiani R.T.D., Setiabudiawan B. (2022). Exploring alternative cytokines as potential biomarkers for latent tuberculosis infection in pregnant women. PLoS ONE.

[31] Vos W.A.J.W., Navas A., Meeder E.M.G., Blaauw M.J.T., Groenendijk A.L., van Eekeren L.E., Otten T., Vadaq N., Matzaraki V., van Cranenbroek B. (2024). HIV immunological non-responders are characterized by extensive immunosenescence and impaired lymphocyte cytokine production capacity. Front. Immunol..

[32] Indrati A.R., Sumarpo A., Haryanto J., Rosmiati N.M.D., Munaya S., Turbawaty D.K., Wisaksana R. (2024). Identification of cytokine signatures in HIV-infected individuals with and without Mycobacterium tuberculosis co-infection. Biomed. Rep..

[33] Serdar C.C., Cihan M., Yücel D., Serdar M.A. (2021). Sample size, power and effect size revisited: Simplified and practical approaches in pre-clinical, clinical and laboratory studies. Biochem. Med..

